# Towards a robust out-of-the-box neural network model for genomic data

**DOI:** 10.1186/s12859-022-04660-8

**Published:** 2022-04-09

**Authors:** Zhaoyi Zhang, Songyang Cheng, Claudia Solis-Lemus

**Affiliations:** 1 Department of Computer Science, University of Wisconsin-Madison, Madison, WI USA; 2 Wisconsin Institute for Discovery, Department of Plant Pathology, University of Wisconsin-Madison, Madison, WI USA

**Keywords:** Generalization error, Phenotype prediction, Convolutional, Natural language processing

## Abstract

**Background:**

The accurate prediction of biological features from genomic data is paramount for precision medicine and sustainable agriculture. For decades, neural network models have been widely popular in fields like computer vision, astrophysics and targeted marketing given their prediction accuracy and their robust performance under big data settings. Yet neural network models have not made a successful transition into the medical and biological world due to the ubiquitous characteristics of biological data such as modest sample sizes, sparsity, and extreme heterogeneity.

**Results:**

Here, we investigate the robustness, generalization potential and prediction accuracy of widely used convolutional neural network and natural language processing models with a variety of heterogeneous genomic datasets. Mainly, recurrent neural network models outperform convolutional neural network models in terms of prediction accuracy, overfitting and transferability across the datasets under study.

**Conclusions:**

While the perspective of a robust out-of-the-box neural network model is out of reach, we identify certain model characteristics that translate well across datasets and could serve as a baseline model for translational researchers.

**Supplementary Information:**

The online version contains supplementary material available at 10.1186/s12859-022-04660-8.

## Background

The ability to accurately predict phenotypes from genomic data is one of the most coveted goals of modern-day medicine and biology. Examples abound: from precision medicine where researchers want to predict a patient’s disease susceptibility based on the genetic information [1–6] to prediction of antibiotic-resistant bacterial strains based on the genomes of pathogenic microbes [7–11]. Examples extend beyond human health into soil and plant health such as the prediction of crops yield (or plant disease susceptibility) based on soil microbiome metagenomic data [12–14] and the prediction of pesticide-resistant microbial strains from plant bacterial pathogen genomes [15–18]. Our ability to anticipate outcomes from data is at our scientific core when we face human disease, environmental challenges, and climate change.

Naturally, biologists and medical researchers have turned to the machine-learning community for answers given the great success of machine-learning methods in a plethora of applications such as computer vision [19, 20] and astrophysics [21, 22] to name a few.

However, the success of machine-learning methods on other fields has not been easily translated to the biological realm [23–26]. Indeed, the complexity of biological omics data has hampered the adoption of machine-learning models, especially neural networks. Among the main challenges of genomic data in neural network models are (1) smaller sample sizes compared to other fields, (2) highly imbalanced datasets, and (3) heterogeneity of training samples and testing samples.

First, despite the advance in high-throughput sequencing technologies, extracting whole genomes remains a time-consuming and expensive task when sample sizes must be in the order of thousands. In addition, data privacy and restrictions on data sharing in medical research restrict scientists’ ability to combine multiple smaller datasets into larger ones suitable for neural network modeling.

Second, more important than sample size, the weak link of deep learning in biological applications is the assumption of homogeneity between training and testing samples. This assumption is violated, for example, in microbial datasets where laboratory samples (training data) and environmentally or clinically collected samples (testing data) can be intricately heterogeneous. This data heterogeneity can cause lack of robustness and generalization errors in neural network models. Robustness is the key ingredient that is needed for neural network models to translate into medical practice and into the phenotype prediction in the agricultural or environmental field.

In literature, there are multiple examples of successfully fit neural network models on biological or medical genomic data [27–34]. However, it remains uncertain whether the proposed models could be translated to other similar datasets with comparable performance. That is, we ask whether the neural network models proposed in literature are robust across heterogeneous (but similar in nature) datasets.

In addition, we approach the existing neural network models with the mindset of a biological or medical user. A biological researcher would see the neural network model in an existing publication and then would try to apply a similar model to their own dataset. First, we explore how easy it is to replicate the analysis on existing publications. Second, by making incremental changes to the model characteristics, we gauge the effect of each model component on the overall performance.

We learn mainly three things: (1) in multiple instances, we are not able to replicate the performance in existing publications either because data is not available, code is not available, or code is corrupted, incomplete or not well-documented; (2) most of the times the good performance of existing models does not translate to alternative datasets, yet we do encounter some model characteristics that are generally robust across datasets and that could serve as a potential baseline model—albeit with modest performance—to start the prediction process from a user perspective, and (3) we find that accurate prediction is a balancing game between underfitting and overfitting, and that small changes in the architecture can have unpredictable outcomes.

The quest for robust neural network models that could tackle the complexities of biological data (and its intrinsic heterogeneity) is imminent. Neural network models cannot be fully applicable in informed patient care, medical or agricultural framework if they cannot guarantee some level of generalization potential given that genomic data are not static but constantly evolving. The difficulty of the prediction problem in biology or medicine is such that it would be naive to believe that there will exist an out-of-the-box model that will be fully transferable (recall the “No Free Lunch” (NFL) theorem [35]: improved performance over one class of problems is offset by performance over another class). Yet, from a user perspective, it is desirable to know if there are certain model characteristics that perform modestly under scrutiny from a variety of different datasets.

While we advise biological or medical users against using out-of-the-box strategies, we conclude from our study that recurrent neural networks are relatively robust across genomics datasets and generally not affected by the size or type of the data. Overfitting is an issue on more complex CNN models (as expected), but it is relatively controlled via regularization schemes. We also found that a general LSTM layer for embedding performed relatively well across datasets and outperforms more intuitive data encoding schemes like doc2vec which performs poorly on all the scenarios we tested. Finally, our work raises awareness to the importance of reproducibility and replicability. As machine-learning scientists, it is crucial to accompany our work with reproducible scripts that are relatively easy to follow by the scientific community so that our findings have an impact across fields, in particular, into the biological and medical community.

## Methods

We focus on convolutional neural networks (CNN) [30, 31, 33, 34] and natural language processing (NLP) [32, 36, 37] on three datasets of increasing size from the available ones in the papers under study (Table 1) and described below.

**Table 1:** Datasets used to test the neural network models

Dataset	Sample size	Sequence Length (bp)	References
Splice	3190	60	[30]
Histone	14965	500	[30]
Motif discovery	269100	101	[31]

*Splice data* In [30], the authors included a splice dataset (also in the UCI machine learning repository [38]). Splice junctions are points on a DNA sequence at which superfluous DNA is removed during the process of protein creation in higher organisms. This dataset has 3190 sequences of length 60 bp and are classified into three classes: exon/intron boundaries (EI: 24%), intron/exon boundaries (IE: 24%), and non-splice (N: 52%).

*Histone data* In [30], the authors included 10 datasets about DNA sequences wrapping around histone proteins. We focus on the H3 occupancy from the histone dataset that has 14,965 sequences of length 500 bp. The H3 indicates the histone type, and the dataset has two classes: the positive class includes DNA sequences that contain regions wrapping around histone proteins (51%) and the negative class does not contain such regions (49%).

*Motif discovery data* In [31], the authors included two ChIP-seq datasets: motif discovery and motif occupancy. These datasets contain the labels of the binding affinity of transcription factors to DNA sequence in 690 different ChIP-seq experiments. We only focus on a subset of 269,100 sequences from the motif discovery data (out of 20,464,149) of length 101 bp. The dataset contains two classes: positive class includes DNA sequences that are motif (50%) and negative class includes DNA sequences that are not motif (50%).

*Data splitting* For all CNN models, we use the following split of the data. The splice dataset is split into 75% for training and 25% for testing with 15% of training data used for validation. The histone dataset is split into 70% for training, 15% for validation, and 15% for testing. The motif discovery data is split into 48.7% for training, 2.6% for validation, and 48.7% for testing. We note that the data partition for the motif discovery dataset deviates from the standard 70-15-15 or 75-25 data partitions. The rationale for this data partition is that the motif discovery data was stored in 690 different files each with a different number of sequences. Given that we do not know how these datasets were created, we wanted to have a uniform representation from all datasets in the training process. The smallest file had 190 sequences, so we randomly selected 190 sequences for each of the 690 files to be used in training. This represents 48.7% of samples used for training. A higher proportion of training samples would imply that some files would be over-represented which could introduce unintended biases in prediction. We choose the same proportion for testing to be able to evaluate the model better given the high heterogeneity of the data leaving only 2.6% for validation.

Data encoding differs for the CNN models and the NLP models, so we describe the encoding procedure in the next sections for each type of model.

### Convolutional neural networks

We test the performance of four convolutional neural network (CNN) models found in literature [30, 31, 33, 34] that have been successful on genomic-based prediction. We assess their performance on their own datasets (when available) and on alternative similar datasets, as well as under incremental modifications of the model characteristics such as data encoding, window size and number of layers. See Table 2 for a summary of the performance tests and models. For all models, we use the cross-entropy loss.

**Table 2:** CNN models along with the datasets used and the performance tests

	Model	Own datasets	Outside datasets	Performance tests
CNN-Nguyen [30]	Fig. 1	Splice, histone	Motif discovery	Number of layers, dimension
CNN-Zeng [31]	Fig. 3	Motif discovery	Splice, histone	Number of layers
DeepDBP [34]	Fig. 4		Splice, histone, motif Discovery	
DeepRAM [33]	Figs. 5, 6, 7		Splice, histone, motif discovery	Data Encoding

#### CNN-Nguyen model [30].

**Fig. 1:**
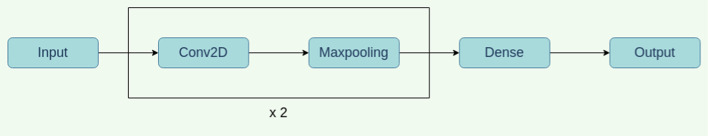
Original CNN-Nguyen (2D) [30]. The dense layer has a dropout rate of 0.5 to prevent overfitting

**Fig. 2:**
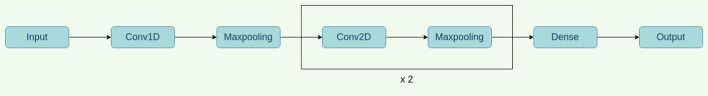
Modified CNN-Nguyen (2D+1D) [30]. The dense layer has a dropout rate of 0.5 to prevent overfitting

We implement the simple neural network in [30] as our baseline model (Fig. 1). The model contains two 2D convolutional layers, each followed by a pooling layer, then the output of the convolutional layers are connected to a fully connected layers. The fully connected layer has a dropout rate of 0.5 to reduce the effect of overfitting. Finally, we use a softmax layer to predict the labels of the input sequences. We denote this original model as CNN-Nguyen2D in the results. In addition to this model, we construct a new model with an extra 1D convolutional layer denoted CNN-Nguyen2D+1D for performance comparison. We compare the performance of the model with a different dimension (1D) and increasing number of layers (Fig. 2). For splice and histone datasets, the batch size is 32 and for motif discovery dataset, the batch size is 512. Kernel size is (3, 3) for all 2D convolutional layers and (1, 3) for the 1D layer in CNN-Nguyen2D+1D. The number of filters in convolutional layer doubles each time we add a new set of these layers (e.g. 16 filters in the first convolutional layer, 32 in the second, 64 in the third). We use the Adam optimizer with learning rate 0.001 and train for 50 epochs which was assessed to allow sufficient training time for convergence (lack of change in loss over the last few epochs) on all datasets. Early Stopping Callback is not used when training these models as convergence was easily assessed in these cases by studying the loss dynamics.

#### CNN-Zeng model [31].

**Fig. 3:**
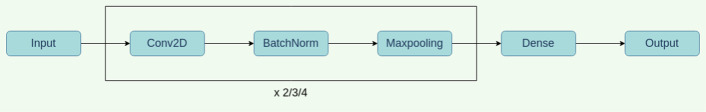
Original CNN-Zeng (2 layers), modified (3,4 layers) [31]. The dense layer has a dropout rate of 0.5 to prevent overfitting

We implement the neural network model in [31] (Fig. 3) that contains two 2D convolutional layers each followed by a batch-normalization and max-pooling layer. The output of the convolutional layers is connected to a fully connected layer. This layer has a dropout rate of 0.5 to prevent overfitting. Finally, a softmax layer is used to predict the class of the input sequence. We denote the original model as CNN-Zeng2 in the results because it has two 2D convolutional layers. We create two new model extensions: CNN-Zeng3 and CNN-Zeng4 with three and four 2D convolutional layers respectively. To explore the effect of the number of layers, we add 2D convolutional, batch-normalization, and max-pooling layers to the end of the convolutional network (Fig. 3). For splice and histone datasets, the batch size is 32 and for motif discovery dataset, the batch size is 512. Kernel size is (3, 3) for all 2D convolutional layers. The number of filters in convolutional layer doubles each time we add a new set of these layers (e.g. 16 filters in the first convolutional layer, 32 in the second, 64 in the third). We use the Adam optimizer with learning rate 0.001 and train for 50 epochs which was assessed to allow sufficient training time for convergence (lack of change in loss over the last few epochs) on all datasets. Early Stopping Callback is not used when training these models as convergence was easily assessed in these cases by studying the loss dynamics.

#### DeepDBP model [34].

**Fig. 4:**
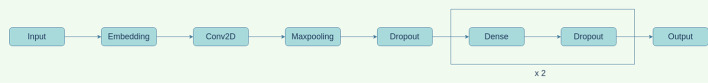
DeepDBP [34]

Even though the source code of this paper is not well structured and contains many different models that are not properly documented, we implement a model based on the paper description which is what a domain scientist (like a biomedical researcher) would do. The model architecture contains a embedding layer, a convolutional layer, max-pooling layer, followed by fully connected layers and the output layer (Fig. 4). For splice and histone datasets, the batch size is 32 and for motif discovery dataset, the batch size is 512. Kernel size is (1, 3) for the 1D convolutional layer with 128 filters. We use the Adam optimizer with learning rate 0.001 and train for 50 epochs which was assessed to allow sufficient training time for convergence (lack of change in loss over the last few epochs) on all datasets. Early Stopping Callback is not used when training these models as convergence was easily assessed in these cases by studying the loss dynamics. Unlike CNN-Nguyen, CNN-Zeng and DeepRAM which only have one dropout layer at the dense layer, the DeepDBP model has three dropout layers: dropout – dense – dropout – dense – dropout – dense (output) with dropout rate of 0.3.

#### DeepRAM model [33].

**Fig. 5:**
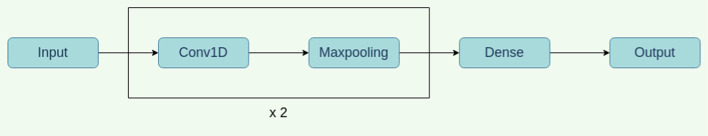
DeepRAM-CNN [33]. The dense layer has a dropout rate of 0.5 to prevent overfitting

**Fig. 6:**
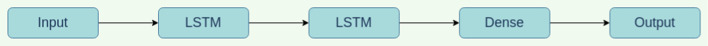
DeepRAM-RNN [33]. The dense layer has a dropout rate of 0.5 to prevent overfitting

**Fig. 7:**
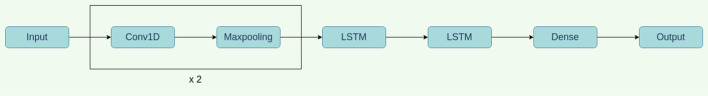
DeepRAM-CNN-RNN [33]. The dense layer has a dropout rate of 0.5 to prevent overfitting

We implement the three models in [33]: 1D convolutional neural networks (Fig. 5) denoted DeepRAM-CNN, recurrent neural networks (Fig. 6) denoted DeepRAM-RNN, and a mixture of 1D convolutional and recurrent neural networks (Fig. 7) denoted DeepRAM-CNN-RNN. For convolutional neural networks, we use two 1D convolutional layers, each followed by a max-pooling layer, and finally fully connected layer (with a dropout rate of 0.5 to prevent overfitting) and output layer. For splice and histone datasets, the batch size is 32 and for motif discovery dataset, the batch size is 512. Kernel size is (1, 3) for the 1D convolutional layer. The number of filters in convolutional layer doubles each time we add a new set of these layers (e.g. 16 filters in the first convolutional layer, 32 in the second). We use the Adam optimizer with learning rate 0.001 and train for 50 epochs which was assessed to allow sufficient training time for convergence (lack of change in loss over the last few epochs) on all datasets. Early Stopping Callback is not used when training these models as convergence was easily assessed in these cases by studying the loss dynamics. For recurrent neural networks, we use two Long Short-Term Memory (LSTM) layers followed by fully connected layers and output layer. For the hybrid neural networks, we use two 1D convolutional layers and two LSTM layers, and finally fully connected layers and output layers. We note that the DeepRAM-RNN and the DeepRAM-CNN-RNN models are not entirely CNN models and share many characteristics with the Natural Language Processing (NLP) models we will describe next. However, we present these models in this section given that they are all part of the DeepRAM paper [33] and we follow the comparisons and analyses highlighted in this work. We compare the changes in performance based on the data encoding as well as comparing the performance of convolutional vs recurrent models.

#### Data encoding

For the first three models (CNN-Nguyen, CNN-Zeng and DeepDBP), we use the same data encoding as in [30] described next. A sliding window of fixed size *k* allows us to traverse the sequence focusing on windows of length *k*. The window of length *k* is a sequence of *k* nucleotides denoted k-mer. The slide stride is how many nucleotides the window moves to the right as it is traversing the sequence. At each step, a k-mer is read from the sequence and added to the k-mer sequence. For example, if a sequence looks like “ACTGG”, a window size of 3 with slide stride of 1 would produce the 3-mers [“ACT”, “CTG”, “TGG”]. The process is similar to how n-grams are created from text with the k-mer being the word and *k* being the “word size”. After that, one-hot encoding is applied to the k-mers. To also include to spatial information of the sequences, we concatenate the encoding of k-mers within a fixed region size. For example, for the 3-mers [“ACT”, “CTG”, “TGG”], a region of 2 would imply that we concatenate [“ACT”, “CTG”] to build the 2D encoded data matrix (see Fig. 4 in [30] for more details). As in [30], we choose a window size of 3 with slide stride of 1 and a region of 2.

For the DeepRAM models, we experiment with two different ways of encoding the sequences. One way is, as described before, to use one-hot encoding with word size 3 and region size 2. The other way is to convert sequences into overlapping k-mers, and embed k-mers into dense vectors using embedding layers. Note that this embedding is different from the one used in the NLP models (described in next section) because of the unit used for encoding. Here, we use the k-mer as the unit for encoding while in NLP models described next, we use the nucleotide as the unit.

### Natural language processing in conjuction with neural networks models for prediction

Traditionally, genomic data is stored as a collection of long strings comprised of the four nucleotides: A,C,G,T. It is thus intuitive to turn to Natural Language Processing (NLP) theory for solid ways to embed the sequences in a latent space. Furthermore, NLP methods naturally overcome one of the main drawbacks of CNN models which is the sparsity of the input vectors.

Here, we focus on two widely used NLP tools: doc2vec [36, 39] and Long Short Term Memory (LSTM) [32, 39, 40]. Both methods share the same objective: represent the input sequence with a low dimensional dense vector yet the specifics differ as is explained below.

**Fig. 8:**
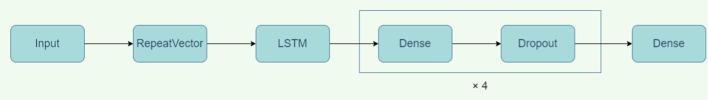
Simple NN for prediction after LSTM-AE (encoder) and doc2vec

We first clarify that the NLP methods are not performing prediction (as the CNN models). Since the purpose of this work is to compare the performance of neural network models on the prediction of phenotypes from genomes, we need to add a neural network model to the NLP model that will perform the prediction of labels (Fig. 8). Table 3 presents a summary of the performance tests and models.

**Table 3:** NLP models along with the datasets used and the performance tests

	Model	Own datasets	Outside datasets	Performance tests
LSTM-layer	Fig. 9		splice, histone, motif discovery	optimizer
doc2vec+NN [36, 39]	Fig. 8		splice, histone, motif discovery	embedding size
LSTM-AE+NN [32]	Figs. 8, 10		splice, histone, motif discovery	batch size

#### LSTM-layer model

**Fig. 9:**
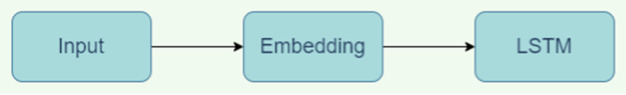
LSTM-layer

We implement the neural network model (Fig. 9) that contains, after the input layer, an embedding layer followed by an LSTM layer with size of 30 for both datasets. There are four dense layers with size decaying by a factor of 2 (128-64-32-16). There is one dropout layer between any two dense layers with dropout rate of 0.2. With this model, we study the changes in performance when using different optimizers: Adam and SGD. For the Adam optimizer, we use a learning rate of 0.001 and for SGD optimizer, we used a learning rate of 0.01. We use Early Stopping Callback on both Adam and SGD optimizers with a maximum number of epochs set at 4000 for the splice and the histone data, and 200 for the motif discovery data. The patience parameter (the threshold to stop the training if the loss stops decreasing further after a certain number of epochs) is set at 100 for both optimizers for the splice data, at 100 and 400 for Adam and SGD respectively for the histone data, and at 10 for both optimizers for the motif discovery data. Changes in the patience parameter are due to differences in speed between the two optimizers when training. For the splice data, training with the Adam optimizer stopped early at 289 epochs while training with SGD optimizer stopped early at 2872 epochs. For the histone data, training with the Adam optimizer stopped early at 154 epochs and at 526 for the SGD optimizer. Finally, for the motif discovery data, training stopped early at 51 epochs for the Adam optimizer, but training reached the maximum number of epochs allowed (200) for the SGD optimizer bringing into question the convergence of such training.

#### doc2vec+NN model [36, 39]

The nature of the doc2vec sequence representation as a semantic vector preserves similarity of sequences in terms of frequency and location of n-grams. We apply the distributed memory mode (DV-PM) as in [36, 39], and then, we use the simple fully connected neural network in Fig. 8 containing two dense layer with size shrinking by a factor of 2 with a dropout layer in between. We study the effect of embedding size in the performance of the model. For all instances of this model, we use the SGD optimizer with learning rate of 0.01 and momentum of 0.9. We use Early Stopping Callback with maximum number of iterations allowed as 1000 for the splice and histone data, and 400 for the motif discovery data. The patience parameter is set at 50 for the splice data, 30 for the histone data and 10 for the motif discovery data. Again, changes in the maximum number of iterations and patience parameter are due to the speed of training for different sample sizes. Training stopped early on all instances of the model. For the splice data, training stopped early at 119, 143, 166, and 264 epochs for the four embedding sizes used (50, 100, 150, and 200). For the histone data, training stopped early at 114, 177, 61, and 118 for the four embedding sizes used (50, 100, 150, and 200). For the motif discovery data, training stopped early at 36, 39, 58, and 11 for the four embedding sizes used (50, 100, 150, and 200).

#### LSTM-AE+NN model [32]

**Fig. 10:**
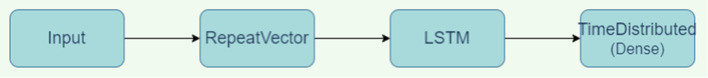
LSTM-AE (encoder) [32]

**Fig. 11:**
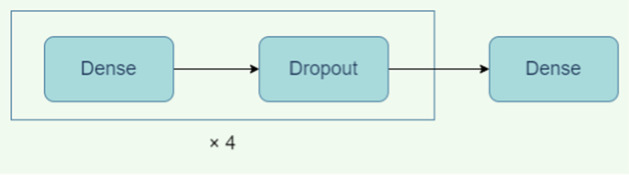
LSTM-AE (decoder) [32]

A LSTM autoencoder model (LSTM-AE) aims to represent a sequence by a dense vector that can be converted back to the original sequence. Indeed, LSTM-AE is comprised of two parts: an encoder network (Fig. 10) that compresses the original sequence into a low dimensional dense vector, and a decoder network (Fig. 11) that converts the vector back to the original sequence. The encoder reads as input an encoded DNA sequence and outputs a dense vector as the embedding for this sequence whose length is a hyper parameter to tune. The decoder reads as input the dense vector produced by the encoder and produces a reconstructed sequence. The accuracy of the autoencoder is measured by comparing the reconstructed sequence to the original sequence. We implement a LSTM-AE following [32] based on the description in their publication given that no reproducible script was available. The LSTM-AE model is trained to achieve maximum reconstruction accuracy of the sequences. Then, since LSTM-AE is not performing classification, we add a simple fully connected neural network (Fig. 8) containing two dense layer with size shrinking by a factor of 2 with a dropout layer in between for the prediction of class labels. The size of the first dense layer is adjusted, as a rule of thumb, to match 1 to 4 times the embedding dimension. We denote this model as LSTM-AE+NN. We note that only the weights corresponding to the simple fully connected neural network are optimized for classification which is different to the LSTM-layer model whose embedding indeed changes during training. We highlight that the LSTM-layer and LSTM-AE models differ on how an embedding is evaluated. The embedding produced by the LSTM-layer model aims to better classify the sequences into the right category while the embedding produced by the LSTM-AE model aims to better capture the sequence itself. We study the effect of the batch size in the performance of the model. For the LSTM-AE training, we use the Adam optimizer with learning rate of 0.001 while for the training of the simple NN for prediction, we use the SGD optimizer with momentum of 0.9 and with learning rate of 0.01 for the splice and motif discovery data, and 0.001 for the histone data. We use Early Stopping Callback on all cases with 2000, 4000 and 200 maximum iterations allowed for splice, histone and motif discovery data respectively for the LSTM-AE training, and 1000, 1500 and 500 maximum iterations allowed for splice, histone and motif discovery data respectively for the simple NN training. In terms of the patience parameter, we set it at 100, 200 and 10 for the splice, histone and motif discovery data respectively for both LSTM-AE and simple NN training. The LSTM-AE training stopped early in almost all cases: (1) for the splice data, at epoch 1474, 424, and 1005 for the three batch sizes used (32, 256, and 1024 respectively); (2) for the histone data, at epoch 549, 422, and 646 for the three batch sizes used (32, 256, and 1024 respectively), and (3) for the motif discovery data, at epoch 78 and 195 for batch sizes 32 and 256. For this data, training reached the maximum number of iterations allowed (200) for the case of batch size of 1024 bringing into question the convergence of this case. The training of the simple NN stopped early in all cases: (1) for the splice data, at epoch 123, 114, and 200 for the three batch sizes used (32, 256, and 1024 respectively); (2) for the histone data, at epoch 212, 805, and 999 for the three batch sizes used (32, 256, and 1024 respectively), and (3) for the motif discovery data, at epoch 185, 146, and 62 for the three batch sizes used (32, 256, and 1024 respectively).

**Table 4:** Training details on NLP models. “Max. It.” means maximum number of iterations allowed

Model	Data	Max. It.	Patience	Early stop	Optimizer
LSTM-AE (32)	Splice	2000	100	1474	Adam (LR=0.001)
LSTM-AE+NN (32)	Splice	1000	100	123	SGD (LR=0.01)
LSTM-AE (256)	Splice	2000	100	424	Adam (LR=0.001)
LSTM-AE+NN (256)	Splice	1000	100	114	SGD (LR=0.01)
LSTM-AE (1024)	Splice	2000	100	1005	Adam (LR=0.001)
LSTM-AE+NN (1024)	Splice	1000	100	200	SGD (LR=0.01)
LSTM-layer	Splice	4000	100	289	Adam (LR=0.001)
LSTM-layer	Splice	4000	100	2872	SGD (LR=0.01)
doc2vec+NN (50)	Splice	1000	50	119	SGD (LR=0.01)
doc2vec+NN (100)	Splice	1000	50	143	SGD (LR=0.01)
doc2vec+NN (150)	Splice	1000	50	166	SGD (LR=0.01)
doc2vec+NN (200)	Splice	1000	50	264	SGD (LR=0.01)
LSTM-AE (32)	Histone	4000	100	549	Adam (LR=0.001)
LSTM-AE+NN (32)	Histone	1000	100	212	SGD (LR=0.001)
LSTM-AE (256)	Histone	4000	200	422	Adam (LR=0.001)
LSTM-AE+NN (256)	Histone	1500	200	805	SGD (LR=0.001)
LSTM-AE (1024)	Histone	4000	200	646	Adam (LR=0.001)
LSTM-AE+NN (1024)	Histone	1500	200	999	SGD (LR=0.001)
LSTM-layer	Histone	3000	100	154	Adam (LR=0.001)
LSTM-layer	Histone	4000	400	526	SGD (LR=0.01)
doc2vec+NN (50)	Histone	1000	30	114	SGD (LR=0.01)
doc2vec+NN (100)	Histone	1000	30	177	SGD (LR=0.01)
doc2vec+NN (150)	Histone	1000	30	61	SGD (LR=0.01)
doc2vec+NN (200)	Histone	1000	30	118	SGD (LR=0.01)
LSTM-AE (32)	Motif	200	10	78	Adam (LR=0.001)
LSTM-AE+NN (32)	Motif	500	10	185	SGD (LR=0.01)
LSTM-AE (256)	Motif	200	10	195	Adam (LR=0.001)
LSTM-AE+NN (256)	Motif	500	10	146	SGD (LR=0.01)
LSTM-AE (1024)	Motif	200	10	200	Adam (LR=0.001)
LSTM-AE+NN (1024)	Motif	500	10	62	SGD (LR=0.01)
LSTM-layer	Motif	200	5	51	Adam (LR=0.001)
LSTM-layer	Motif	200	5	200	SGD (LR=0.01)
doc2vec+NN (50)	Motif	400	10	36	SGD (LR=0.01)
doc2vec+NN (100)	Motif	400	10	39	SGD (LR=0.01)
doc2vec+NN (150)	Motif	400	10	58	SGD (LR=0.01)
doc2vec+NN (200)	Motif	400	10	11	SGD (LR=0.01)

We summarize the training details for the NLP models in Table 4. We note that since the training of the CNN was simpler (50 epochs in all cases), we do not need a summarizing table for the training of the CNN models.

#### Data encoding

For the LSTM-layer and the LSTM-AE models, we use the same data encoding as described next. Each nucleotide is converted to a label number. For example, [“A”, “C”, “G”, “T”], are encoded as [3, 2, 1, 0] in descending lexicographical order. The LSTM-layer is crucial given the intractable growth in dimension of the input vector. That is, a sequence containing 6000 nucleotides would be represented by a sequence of 6000 numbers. For the doc2vec model, we encode the sequences based on 3-mers with slide stride of 1. For example, for the “ACTGG” sequence, the 3-mers are [“ACT”, “CTG”, “TGG”]. We construct a dictionary with all the 3-mers in the training set. While it is unlikely for 3-mers in the test set to not appear in the dictionary, we categorize these instances as out-of-vocabulary (OOV) with a unique encoding.

## Results

### The role of dimension and number of layers on CNN

**Fig. 12:**
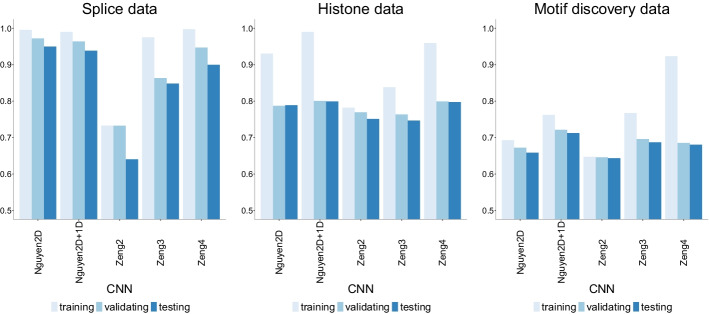
Accuracy of CNN models of increasing number of layers on three datasets of increasing size. Nguyen2D corresponds to the original CNN model in [30], while Nguyen2D+1D corresponds to the same model with an extra 1D convolutional layer. Similarly, Zeng2 corresponds to the original model in [31] which has two 2D convolutional layers while Zeng3 and Zeng4 correspond to models with three and four 2D convolutional layers respectively. There is an inverse relationship between accuracy and data size with the largest dataset (motif discovery) having the lowest accuracy overall. Adding one layer with different dimension (CNN-Nguyen2D+1D) improves the accuracy slightly, but more layers of the same dimension (CNN-Zeng3 with 3 layers and CNN-Zeng4 with 4 layers) only increase the overfitting

Figure 12 shows the training, validating and testing accuracy of the CNN models when varying the number of layers for the three datasets. Nguyen2D corresponds to the original CNN model in [30], while Nguyen2D+1D corresponds to the same model with an extra 1D convolutional layer. Similarly, Zeng2 corresponds to the original model in [31] which has two 2D convolutional layers while Zeng3 and Zeng4 correspond to models with three and four 2D convolutional layers respectively.

For the smallest dataset (splice), all models have a testing accuracy higher than 80% which is similar to what is reported in the original CNN-Nguyen paper [30] (88.9%) except for the original model in CNN-Zeng [31]. Adding more layers improves the performance of the CNN-Zeng model, but not the CNN-Nguyen model. There is not any strong evidence of overfitting in any of the models in the splice data.

For the medium size dataset (histone), all models have a similar testing accuracy (slightly below 80%). In this dataset, adding more layers merely increases the training accuracy and thus, the overfitting. Lastly, for the largest dataset (motif discovery), all testing accuracies are below 70%. There is a slight improvement in the CNN-Nguyen model when adding one more 1D layer, yet for the case of CNN-Zeng, more layers only increase the training accuracy and thus, the overfitting. We compare the performance of the CNN-Zeng model with and without regularization in the Additional file 1: Appendix.

To sum up, accuracy decreases with data size with the largest data having the lowest reported accuracy. In addition, adding more layers to a CNN model does increase accuracy for smaller datasets, but it appears to only increase overfitting on larger datasets. This assertion is counterintuitive as overfitting is thought to be the result of parameter-rich models on small size data. In our analyses, overfitting indeed appears as a result of more complex models (more layers) yet only on larger datasets. It is important to note that this atypical performance could be due to the distinct data partition chosen for the motif discovery data (48-3-48 in contrast to a standard 70-15-15). For this dataset, we prioritized a equal contribution to the training samples from each of the 690 input files in order to prevent unintended bias in predictions caused by heterogeneity in the sequences. This choice is not meant to be perfect and can create another set of complications (e.g., the unexpected decreased accuracy). Future work should address implications in prediction due to data partition choices when faced with highly heterogeneous datasets.

**Fig. 13:**
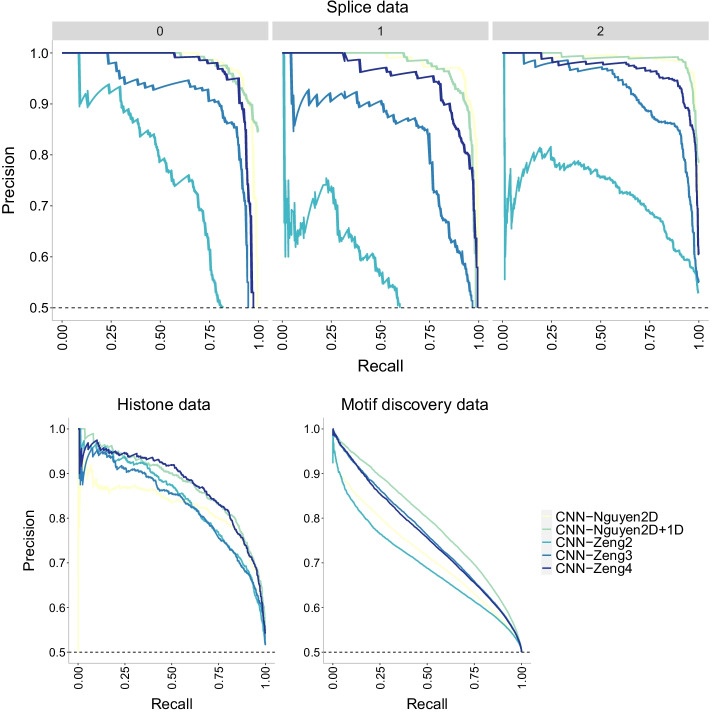
Precision-recall curves of CNN models of increasing number of layers on the three datasets of increasing size. The higher the curve, the better performance with a horizontal dashed line to represent random prediction. An ideal precision-recall curve would cross the (1,1) point. The splice data has three panels since precision-recall curves assume binary classification and the splice dataset has three classes (0, 1, 2). Each panel corresponds to prediction one class vs the other two combined. Nguyen2D corresponds to the original CNN model in [30], while Nguyen2D+1D corresponds to the same model with an extra 1D convolutional layer. Similarly, Zeng2 corresponds to the original model in [31] which has two 2D convolutional layers while Zeng3 and Zeng4 correspond to models with three and four 2D convolutional layers respectively. The CNN-Nguyen models outperform the other models across datasets

**Fig. 14:**
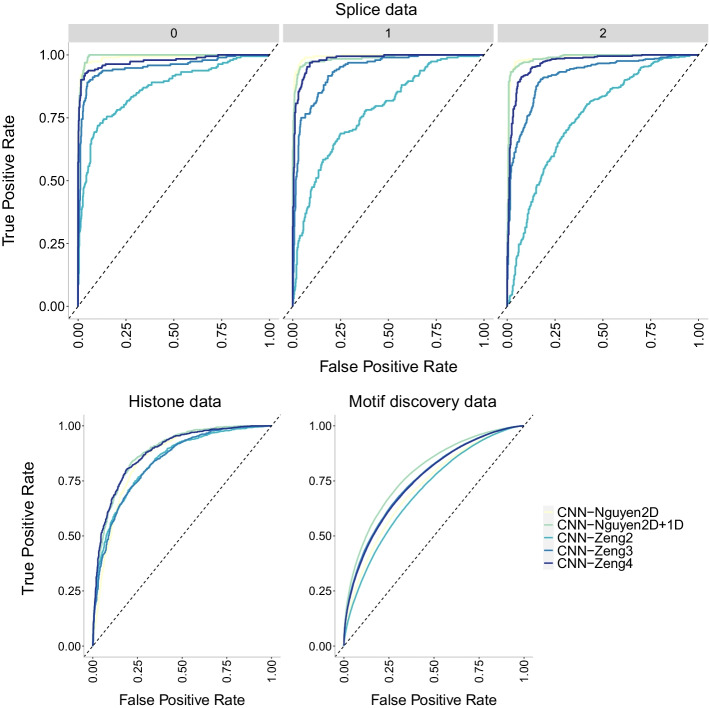
ROC curves of CNN models of increasing number of layers on the three datasets of increasing size. The higher the curve, the better performance with a 45$^{\circ }$ dashed line to represent random prediction. The splice data has three panels since ROC curves assume binary classification and the splice dataset has three classes (0, 1, 2). Each panel corresponds to prediction one class vs the other two combined. Nguyen2D corresponds to the original CNN model in [30], while Nguyen2D+1D corresponds to the same model with an extra 1D convolutional layer. Similarly, Zeng2 corresponds to the original model in [31] which has two 2D convolutional layers while Zeng3 and Zeng4 correspond to models with three and four 2D convolutional layers respectively. The CNN-Nguyen models outperform the other models across datasets

Finally, we investigate the precision-recall curves of the models in Fig. 13. The CNN-Nguyen models outperform those in CNN-Zeng across datasets with the original CNN-Zeng2 displaying the worse performance. This behavior is confirmed with the ROC curves in Fig. 14.

### The role of data encoding

**Fig. 15:**
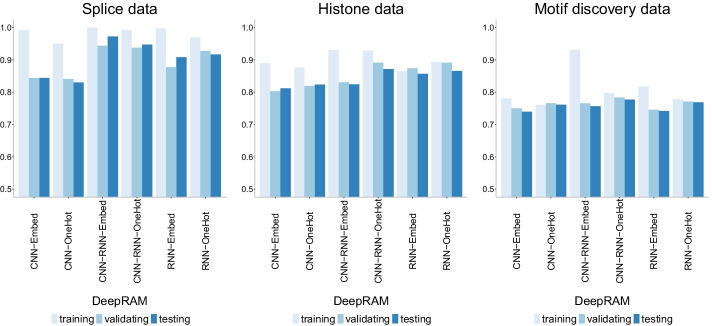
Accuracy of DeepRAM models [33] with two different data encoding schemes: one-hot encoding (OneHot) and embedding layer (Embed) on three datasets of increasing size. CNN corresponds to the convolutional model, RNN corresponds to the recurrent model and CNN-RNN corresponds to the combined model. Accuracy decreases with data size, and all models display a similar behavior on the different data encoding schemes

**Fig. 16:**
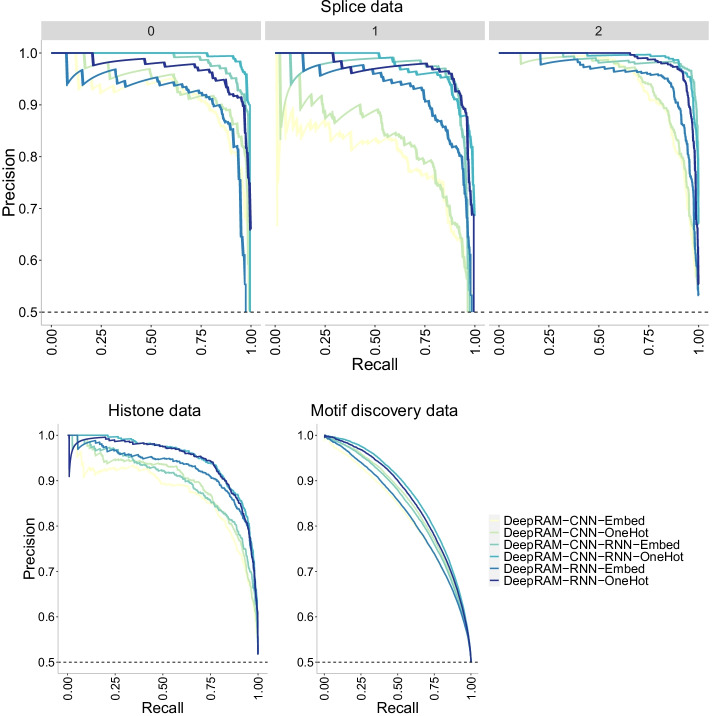
Precision-recall curves of DeepRAM models [33] with two different data encoding schemes: one-hot encoding (OneHot) and embedding layer (Embed) on three datasets of increasing size. The higher the curve, the better performance with a horizontal dashed line to represent random prediction. An ideal precision-recall curve would cross the (1,1) point. The splice data has three panels since precision-recall curves assume binary classification and the splice dataset has three classes (0, 1, 2). Each panel corresponds to prediction one class vs the other two combined. CNN corresponds to the convolutional model, RNN corresponds to the recurrent model and CNN-RNN corresponds to the combined model. Accuracy decreases with data size, and all models display a similar behavior on the different data encoding schemes

**Fig. 17:**
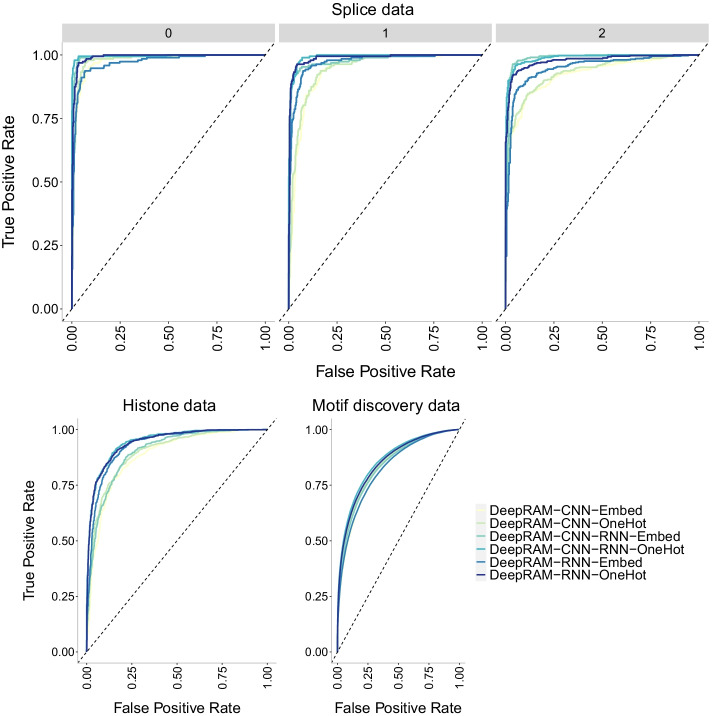
ROC curves of DeepRAM models [33] with two different data encoding schemes: one-hot encoding (OneHot) and embedding layer (Embed) on three datasets of increasing size. The higher the curve, the better performance with a 45$^{\circ }$ dashed line to represent random prediction. The splice data has three panels since ROC curves assume binary classification and the splice dataset has three classes (0, 1, 2). Each panel corresponds to prediction one class vs the other two combined. CNN corresponds to the convolutional model, RNN corresponds to the recurrent model and CNN-RNN corresponds to the combined model. Accuracy decreases with data size, and all models display a similar behavior on the different data encoding schemes

It appears that the type of data encoding (one-hot encoding vs embedding layer) does not have a strong influence on the performance of the DeepRAM models in [33]. Figure 15 shows the accuracy of the models which is lowest overall for the largest dataset (motif discovery) yet there is not a clear difference across models or data encoding types. The combined model (CNN-RNN) seems to slightly outperform the other models and this behavior is also apparent in the precision-recall curves (Fig. 16) and in the ROC curves (Fig. 17). However, care must be taken in that the combined model with embedding layer (CNN-RNN-Embed) seems to overfit in the motif discovery data while the one-hot encoding version of the same model does not show overfitting, so it appears that one-hot encoding should be preferred.

Importantly, the behavior of DeepRAM seems to translate well across datasets. Accuracy lies between 80% and 90% for the smallest dataset (splice) and around 75% for the largest dataset (motif discovery). As a point of comparison, the accuracy presented in original DeepRAM paper [33] ranges from 83.6% to 99.4% on data from 83 ChIP-seq experiments in the ENCODE project.

### The role of the optimizer

**Fig. 18:**
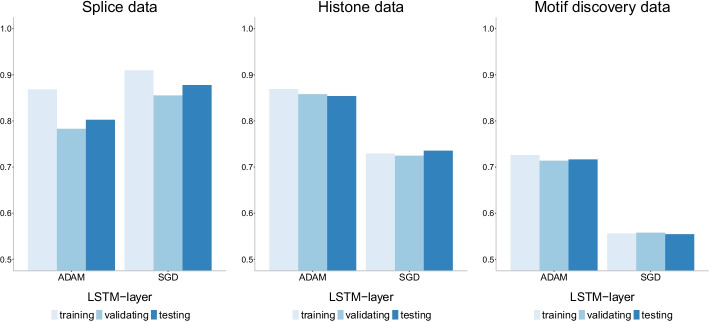
Accuracy of LSTM-layer model with two different optimizers: Adam and SGD on three datasets of increasing size. There is no evidence of overfitting with this model on any of the datasets. In addition, the best optimizer varies with SGD outperforming Adam for the smallest dataset (splice) and Adam outperforming SGD on the other two datasets. It is widely accepted that SGD performs better in terms of finding global optima. However, due to its low speed, it can get stuck in one plateau too long

**Fig. 19:**
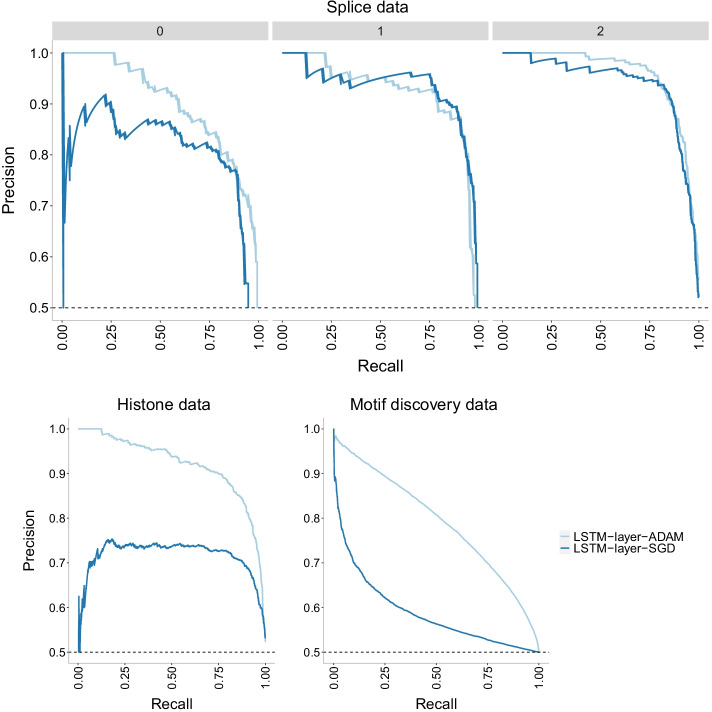
Precision-recall curves of LSTM-layer model with two different optimizers: Adam and SGD on three datasets of increasing size. The higher the curve, the better performance with a horizontal dashed line to represent random prediction. An ideal precision-recall curve would cross the (1,1) point. The splice data has three panels since precision-recall curves assume binary classification and the splice dataset has three classes (0, 1, 2). Each panel corresponds to prediction one class vs the other two combined. The best optimizer varies with SGD outperforming Adam for the smallest dataset (splice) and Adam outperforming SGD on the other two datasets. It is widely accepted that SGD performs better in terms of finding global optima. However, due to its low speed, it can get stuck in one plateau too long

**Fig. 20:**
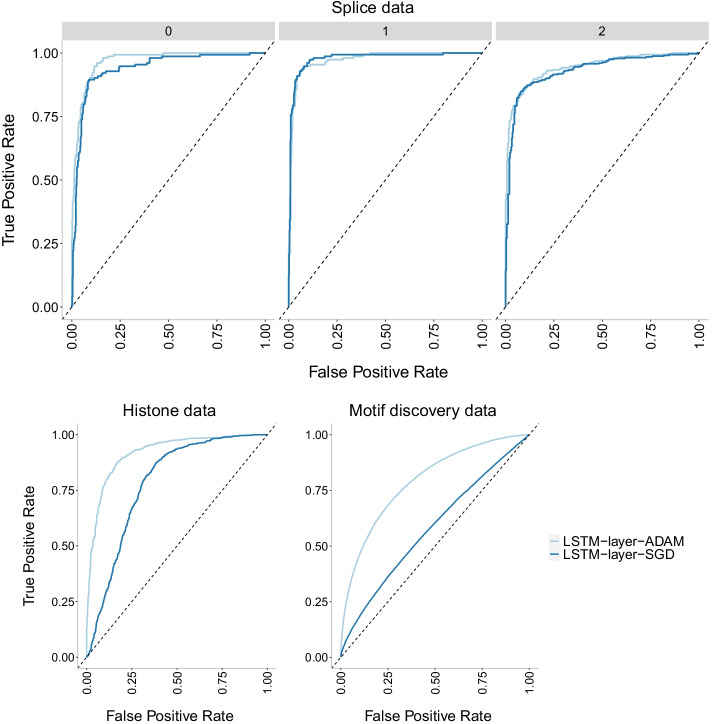
ROC curves of LSTM-layer model with two different optimizers: Adam and SGD on three datasets of increasing size. The higher the curve, the better performance with a 45$^{\circ }$ dashed line to represent random prediction. The splice data has three panels since ROC curves assume binary classification and the splice dataset has three classes (0, 1, 2). Each panel corresponds to prediction one class vs the other two combined. The best optimizer varies with SGD outperforming Adam for the smallest dataset (splice) and Adam outperforming SGD on the other two datasets. It is widely accepted that SGD performs better in terms of finding global optima. However, due to its low speed, it can get stuck in one plateau too long

The SGD optimizer outperforms the Adam optimizer on the LSTM-layer model for the smallest dataset (splice) while Adam outperforms SGD for the two larger datasets (histone and motif discovery). See Fig. 18 for accuracy, Fig. 19 for precision-recall curves and Fig. 20 for ROC curves. While [41] has already discussed the convergence issues of the Adam optimizer, we also need to note that the difference in performance can be due to differences with the Early Stopping Callback patience parameter. It is widely accepted that SGD performs better in terms of finding global optima. However, due to its low speed, it can get stuck in one plateau too long. We note that the comparison of optimizer behavior has ignited multiple studies. For a more comprehensive investigation on the role of optimizers in neural network models, see [42].

### The role of the embedding size

**Fig. 21:**
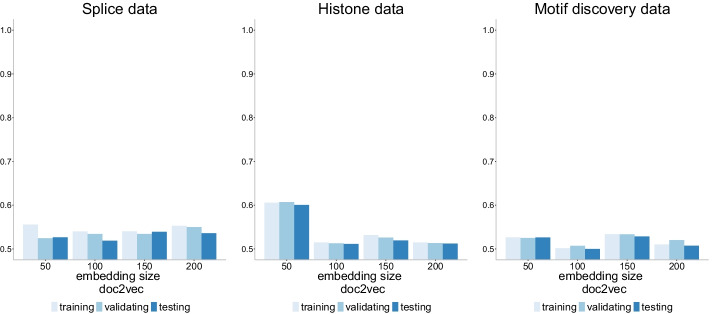
Accuracy of doc2vec+NN model with four different embedding sizes (50, 100, 150, 200) on three datasets of increasing size. The performance is poor in all cases with accuracy barely exceeding 50% across datasets

Of all the neural network models compared in this work, the doc2vec version performs the worse with accuracy barely exceeding 50% (Fig. 21). The size of embedding does not appear to have a strong influence on the accuracy, and if anything, it appears to slightly decrease accuracy as the size of embedding increases for some datasets (e.g. histone). The poor performance of the doc2vec models is evident in the precision-recall curves (Additional file 1: Appendix) and the ROC curves as well (Additional file 1: Appendix). This behavior contradicts the results of the original work of doc2vec on sequences [39] which reported 97% specificity (true negative rate), 93% sensitivity (true positive rate or recall), and 95% accuracy for binary classification (as in histone and motif discovery data) and 83% precision, 81.5% sensitivity, 81% accuracy for multiclass classification (as in splice data). The lack of congruence could be due to lack of robustness of the model across datasets, but more likely can be explained by the length of the sequences. While the original study has an average length of 425 bp with sequences as long as 22,152 bp, the sequences used here have length 60, 101 and 500 bp.

### The role of batch size

**Fig. 22:**
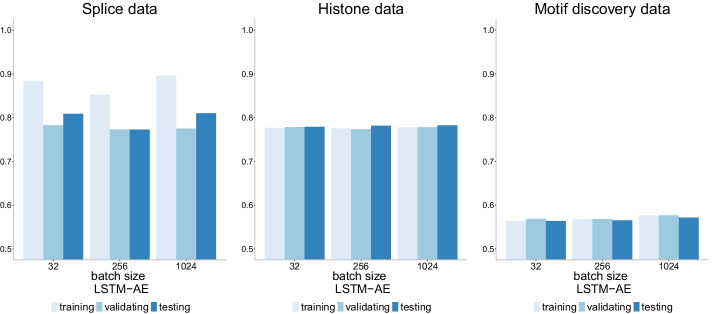
Accuracy of LSTM-AE+NN model [32] with three different batch sizes (32, 256 and 1024) on three datasets of increasing size. There is no evidence of overfitting with this model, and accuracy seems to decrease as the data size increases with the largest data (motif discovery) having the smallest accuracy (barely above 50%)

**Fig. 23:**
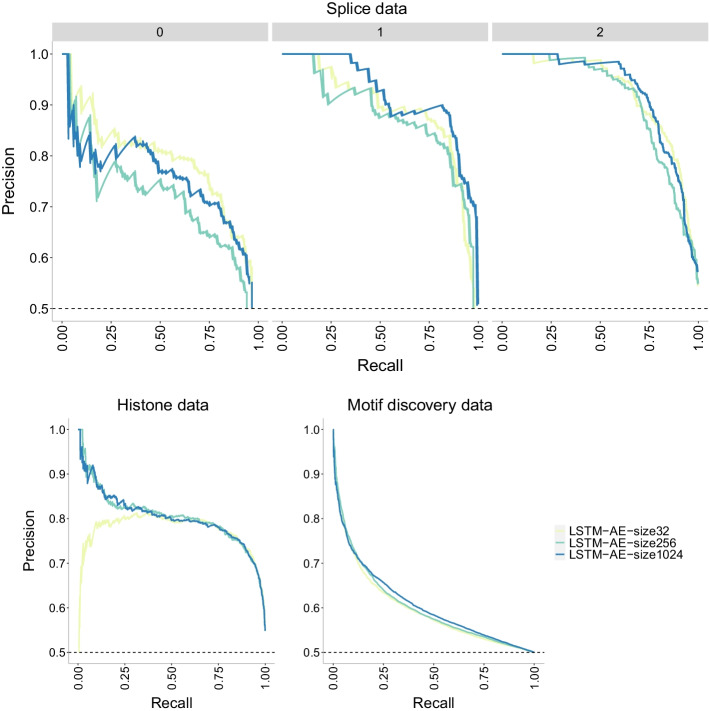
Precision-recall curves of LSTM-AE+NN model [32] with three different batch sizes (32, 256 and 1024) on three datasets of increasing size. The higher the curve, the better performance with a horizontal dashed line to represent random prediction. An ideal precision-recall curve would cross the (1,1) point. The splice data has three panels since precision-recall curves assume binary classification and the splice dataset has three classes (0, 1, 2). Each panel corresponds to prediction one class vs the other two combined. Unlike other models, there is a clear distinction in the class 0 prediction performance of this model compared to other classes in the splice data. It appears that class 0 is harder to predict with a lower recall for a given precision value compared to the other classes

**Fig. 24:**
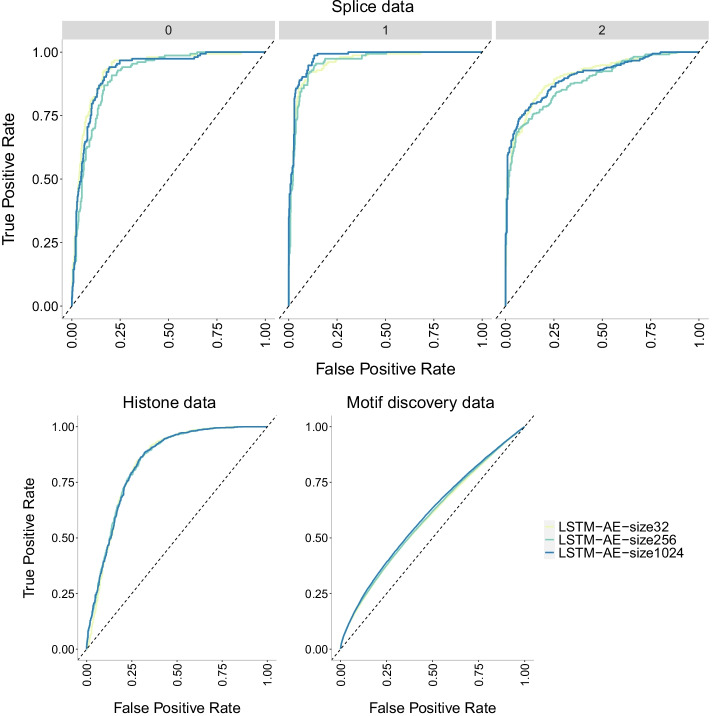
ROC curves of LSTM-AE+NN model [32] with three different batch sizes (32, 256 and 1024) on three datasets of increasing size. The higher the curve, the better performance with a 45$^{\circ }$ dashed line to represent random prediction. The splice data has three panels since ROC curves assume binary classification and the splice dataset has three classes (0, 1, 2). Each panel corresponds to prediction one class vs the other two combined. Again, we see no differences with respect to batch size and ROC curves close to the 45$^{\circ }$ dashed line (random prediction) for the motif discovery data

Batch size has zero impact on the accuracy of the LSTM-AE model [32] (Fig. 22) with all three batch sizes (32, 256 and 1024) showing the same accuracy levels for a given dataset. Accuracy is instead affected by the size of the data with the largest dataset (motif discovery) barely exceeding 50%. Also, this model appears to be robust to overfitting across datasets. The same conclusions can be drawn from the precision-recall curves (Fig. 23) and the ROC curves (Fig. 24). In the precision-recall curve it stands out that the class 0 in the splice data is harder to be predicted with his model compared to the other classes.

### Overall comparison of models

**Table 5:** Models with the highest testing accuracy for each dataset

Model	Splice	Histone	Motif discovery
CNN-Nguyen	Original (two 2D layer)	Extra 1D layer	Extra 1D layer
CNN-Zeng	Four layers L2-reg	Four layers L2-reg	Four layers L2-reg
DeepRAM	RNN-Embed	RNN-OneHot	RNN-OneHot
LSTM-layer	SGD	Adam	Adam
LSTM-AE	Batch size 1024	Batch size 1024	Batch size 1024
doc2vec	Embed size 150	Embed size 50	Embed size 150

Among of all options, we select the models with highest testing accuracy for each of the categories (listed in Table 5): CNN-Nguyen [30], CNN-Zeng [31], DeepRAM [33], doc2vec, LSTM-AE [32] and LSTM-layer and for each of the three datasets (splice, histone and motif discovery). We also add the model in DeepDBP [34] to the comparison.

**Fig. 25:**
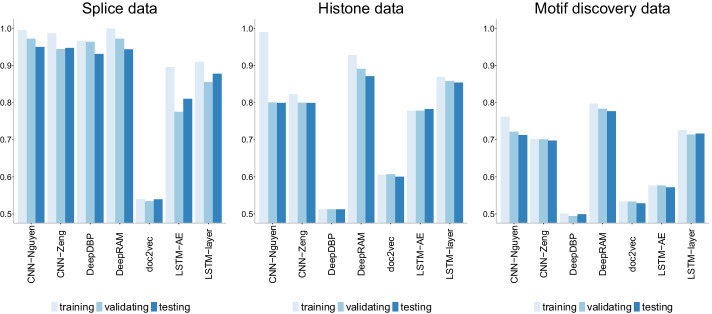
Accuracy of the models with highest testing accuracy among all comparisons (Table 5) across datasets. DeepDBP [34] shows the worst robustness across datasets, while DeepRAM [33] shows both the best accuracy and robustness across datasets. Overfitting does not appear to be an issue except for CNN-Nguyen on the histone data

Regarding accuracy (Fig. 25), first, we note that the behavior of DeepDBP is not robust across datasets with accuracy levels never exceeding 55% for the histone and motif discovery data while the reported accuracy on the original paper [34] was 84.31% for a data of sample size of 1075 sequences. It appears that the performance of DeepDBP is highly dependent on the specifics of the data at hand.

Next, we notice that doc2vec+NN behaves poorly with accuracy levels barely exceeding 50% in all three datasets. We reiterate that this poor performance could be due to the short length of the sequences used here. Overall, DeepRAM outperforms all other models across datasets which has the added strength of robustness given that the accuracy levels are not far from the accuracy levels reported in the original DeepRAM paper [33] (88.9%). Both CNN models (CNN-Nguyen and CNN-Zeng) perform well across datasets albeit less accurately than DeepRAM. Overfitting does not appear to be a relevant factor among these models, except for CNN-Nguyen on the histone data.

**Fig. 26:**
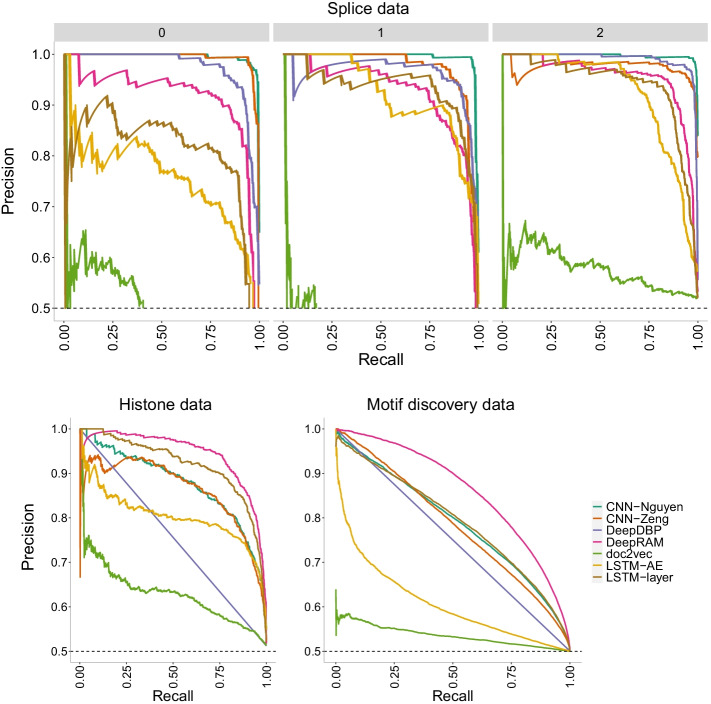
Precision-recall curves of CNN models of increasing number of layers on the three datasets of increasing size. The higher the curve, the better performance with a horizontal dashed line to represent random prediction. An ideal precision-recall curve would cross the (1,1) point. The splice data has three panels since precision-recall curves assume binary classification and the splice dataset has three classes (0, 1, 2). Each panel corresponds to prediction one class vs the other two combined. DeepRAM outperforms all models in the histone and motif discovery datasets, and behaves well on the splice data. The CNN models (Nguyen and Zeng) outperform all models on the splice data

**Fig. 27:**
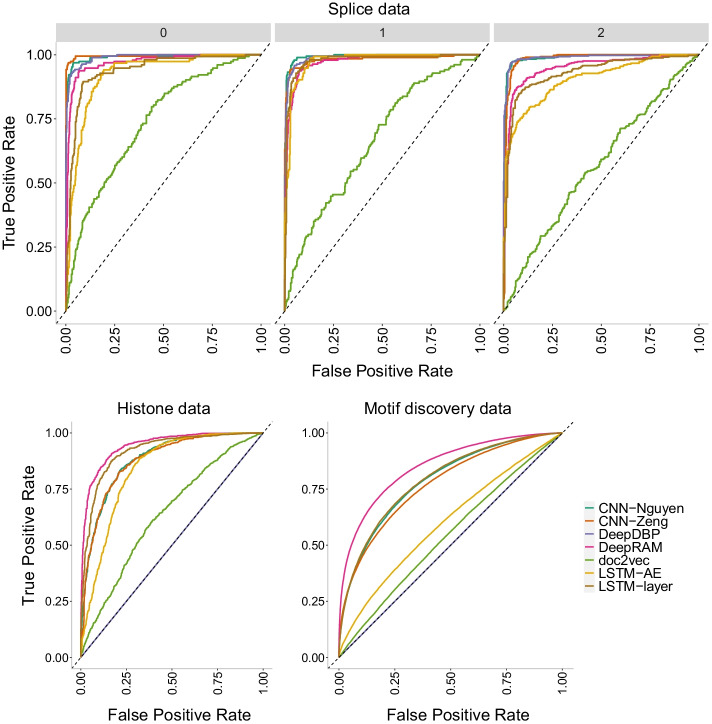
ROC curves of CNN models of increasing number of layers on the three datasets of increasing size. The higher the curve, the better performance with a 45$^{\circ }$ dashed line to represent random prediction. The splice data has three panels since ROC curves assume binary classification and the splice dataset has three classes (0, 1, 2). Each panel corresponds to prediction one class vs the other two combined. DeepRAM outperforms all models in the histone and motif discovery datasets, and behaves well on the splice data. The CNN models (Nguyen and Zeng) outperform all models on the splice data. DeepDBP behaves as a random predictor on the histone and motif discovery data

Figure 26 shows the precision-recall curves where the same conclusions are confirmed with DeepRAM outperforming all models in the histone and motif discovery datasets. The CNN models (CNN-Nguyen and CNN-Zeng) outperform all models for the splice data. We note that prediction of class 0 in the splice data appears to be harder than prediction of the other two classes as evidenced by lower overall curves. The doc2vec model performs poorly on all datasets. Similar conclusions are drawn with the ROC curves (Fig. 27) with DeepDBP behaving as a random predictor on the histone and motif discovery data.

## Discussion

Neural network models provide endless opportunities for prediction and classification in biological applications [43, 44], yet much remains unknown regarding the transferability of the performance across datasets. Robustness across datasets of similar nature is a key ingredient to translate neural network models into medical, agricultural or environmental practice. Here, we study the performance on genomic data of convolutional neural networks (CNN) and neural network models assisted by natural language processing (NLP). We highlight that the conclusions we found are restricted to the datasets and the models selected, and thus, more work is needed to be able to extend conclusions beyond the current study.

We find that DeepRAM outperforms all other models especially the recurrent version (RNN) in terms of prediction accuracy, overfitting, and robustness across datasets. Compared to CNN models (CNN-Nguyen and CNN-Zeng) whose prediction accuracy dramatically decreases with larger datasets, DeepRAM models experience a much smaller accuracy decrease. Furthermore, the accuracy levels of DeepRAM that we find here are comparable to those reported in the original DeepRAM paper [33] and thus, we can conclude that the DeepRAM models are more robust, transferable and generalizable across genomic datasets with varied characteristics. It is interesting to notice that DeepRAM outperforms CNN-Nguyen and CNN-Zeng even when we are using the original datasets in both CNN papers [30, 31].

DeepDBP lacks robustness across datasets at least for the datasets compared in this work. The original paper of DeepDBP [34] reported prediction accuracy levels of 84.31% and while we find a prediction accuracy of around 90% for the splice data, for the histone and motif discovery datasets, the DeepDBP prediction accuracy barely exceeds 50%. Furthermore, the DeepDBP paper did not provide usable reproducible scripts that we could follow, so the poor performance could be due to discrepancies between the model implemented here and the model implemented in the original DeepDBP. Given that our goal was to approach this study from the perspective of a domain scientist (biomedical researcher), we believe that such researcher would read a NN article (like the DeepDBP), and then try to fit such model on their own data. When NN papers provide clear code (python notebooks, for example), they facilitate this task to domain scientists. The authors of DeepDBP, however, did not provide clear code to fit their models, so we test the performance of a model based on the paper description (which is what the biomedical researcher would do). We hope to bring attention to reproducibility practices to help domain scientists fit NN models that appear in literature in their own datasets.

In terms of overfitting, the gap between training and testing accuracy increases as the number of layers increases for CNN models. This behavior is more evident for larger datasets (histone and motif discovery) than smaller datasets (splice). We reiterate that this atypical performance could be due to the choice of data partition into 48.7% training set for the motif discovery data (far from the standard 70-15-15 data partition). While this choice was made in an attempt to reduce bias caused by heterogeneous input sequences, it is far from perfect. Future work should address implications in prediction due to data partition choices when faced with highly heterogeneous datasets.

It is noteworthy that more LSTM layers do not seem to increase overfitting since LSTM-layer (one layer), LSTM-AE (one layer) and DeepRAM-RNN (two layers) have no noticeable overfitting patterns though a more thorough investigation of LSTM layers is still lacking. The only overfitting case for DeepRAM happens on the motif discovery data in the combined model (CNN-RNN) with embedding data encoding. It seems advisable to utilize one-hot encoding for DeepRAM models to prevent the potential of overfitting.

The doc2vec encoding performed poorly on all scenarios. Given that the prediction model for doc2vec and LSTM-AE [32] is the same (the simple NN in Fig. 8) and LSTM-AE dramatically outperforms doc2vec, we do not recommend the use of doc2vec for data embedding and recommend the use of LSTM autoencoders instead. This is especially true for the case of shorter sequences. For the LSTM-AE model, the batch size made no difference in performance and this model seems to be very robust to overfitting, yet we do see smaller accuracy with larger datasets

We conclude by raising awareness to the importance of reproducibility in science. In many instances, it was impossible to replicate the results of existing publications given the lack of reproducible well-documented scripts and available data. Reproducibility is crucial not just for the sake of open science, but to maximize the applicability of our machine-learning findings into a biological or medical community who might not have a strong programming background.

### Practical advice for domain scientists

Among the models here compared, recurrent neural network models (specifically DeepRAM-RNN [33]) outperform convolutional neural network models in terms of prediction accuracy, overfitting and transferability across datasets. More LSTM layers produce higher prediction accuracy without overfitting, unlike more convolutional layers which tend to produce modestly higher accuracy, but also a larger gap between training and testing accuracy. We recommend accompanying extra convolutional layers with regularization. Convolutional neural networks have a reasonable performance overall, but their accuracy is affected by the size of the data with larger (more heterogeneous) datasets having lower prediction accuracy, a behavior not seen with RNN. For data encoding, the intuitive nature of doc2vec does not translate into good prediction performance and less interpretable encoders like LSTM-AE [32] should be preferred, especially for the case of shorter sequences as illustrated in the three datasets here used. The doc2vec encoder followed by a simple NN performs poorly (accuracy barely exceeding 50%) in all tested scenarios unlike LSTM-AE [32] followed by the same simple NN which manages moderately good accuracy (comparable to CNN models) across datasets and without too evident overfitting. In terms of model characteristics, embedding size and batch size do not seem to play any important role in our comparisons, while the optimizer in conjunction with the patience parameter do seem to play a role in the comparisons (see also [42]). We conclude by highlighting that while this work is intended to provide practical advice to domain scientists who are interested in fitting neural network models on their data, it does not intend for domain scientists to work in isolation as nothing can replace the powerful interdisciplinary connections between the domain scientific community and the machine-learning community.

## Supplementary Information

**Additional file 1**. Supplemental figures.

## Data Availability

The data was made publicly available by the original manuscripts. All the scripts developed in this work are publicly available in the GitHub repository https://github.com/solislemuslab/dna-nn-theory.
