# Supplementary material for: Towards a robust out-of-the-box neural network model for genomic data
Source: BMC Bioinformatics. 2022 Apr 9;23:125. doi: 10.1186/s12859-022-04660-8 (PMC8994362; doi:10.1186/s12859-022-04660-8)
Supplement: Supplementary file 1 — Additional file 1. Supplemental figures. [file 12859_2022_4660_MOESM1_ESM.pdf]

Appendix: Towards a robust out-of-the-box neural network model for genomic data

See Figure 1 for the precision-recall plot on the role of the embedding size for the doc2vec model, and see Figure 2 for the ROC curve plot. See Figures 3 and 4 for the effect of regularization on the CNN-Zeng model. Finally, Figures 5 to 9 show the learning dynamics of loss and accuracy for all the models presented in this work.

Reproducible Julia script for random order of first authors

```
using Random
s = 313627913
Random.seed!(s)
people = ["songyang","zhaoyi"]
people[randperm(length(people))]
```

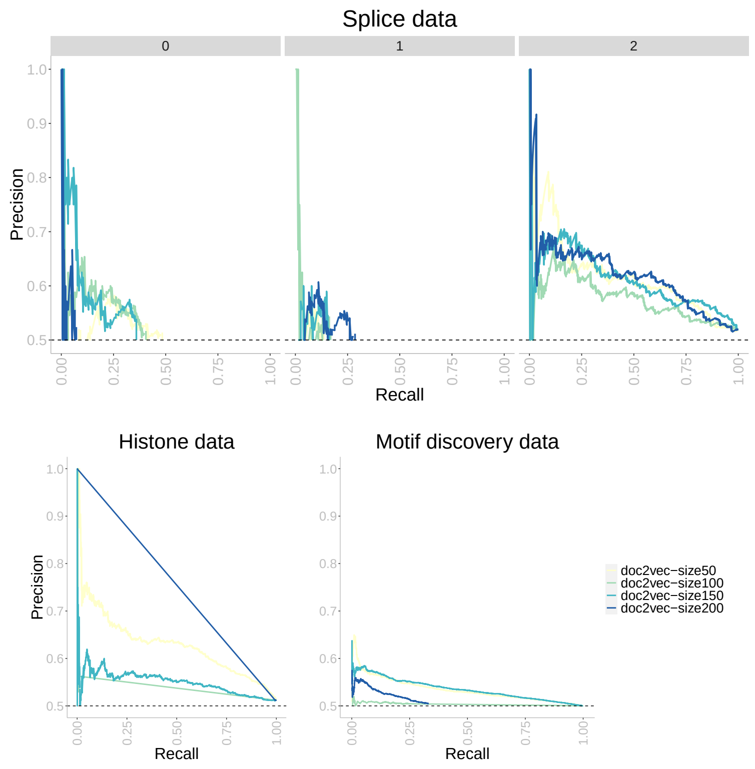

Figure 1 Precision-recall curves of doc2vec+NN model with four different embedding sizes (50, 100, 150, 200) on three datasets of increasing size. The higher the curve, the better performance with a horizontal dashed line to represent random prediction. An ideal precision-recall curve would cross the (1,1) point. The splice data has three panels since precision-recall curves assume binary classification and the splice dataset has three classes (0, 1, 2). Each panel corresponds to prediction one class vs the other two combined. The performance is poor in all cases.

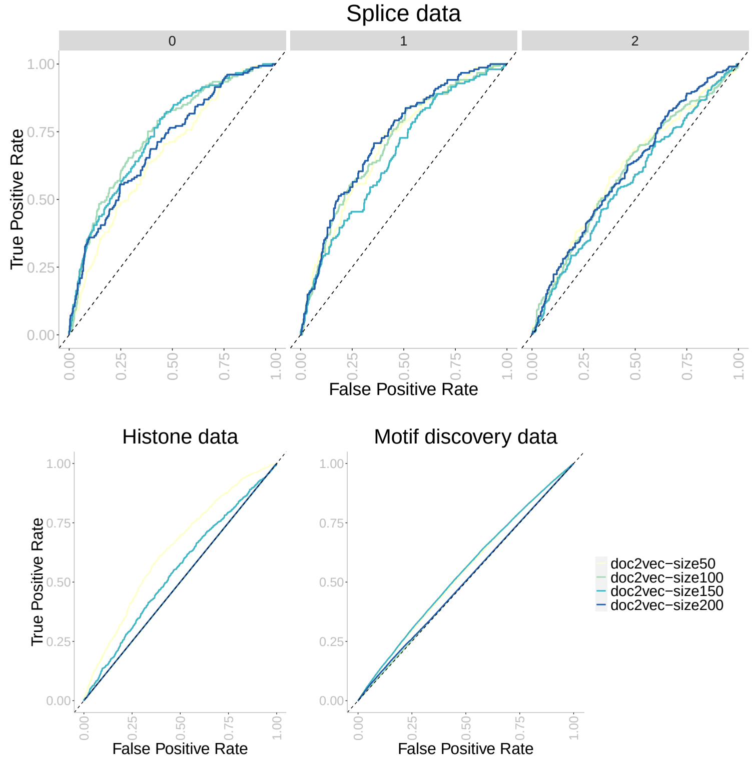

Figure 2 ROC curves of doc2vec+NN model with four different embedding sizes (50, 100, 150, 200) on three datasets of increasing size. The higher the curve, the better performance with a 45° dashed line to represent random prediction. The splice data has three panels since ROC curves assume binary classification and the splice dataset has three classes (0, 1, 2). Each panel corresponds to prediction one class vs the other two combined. The performance is poor in all cases.

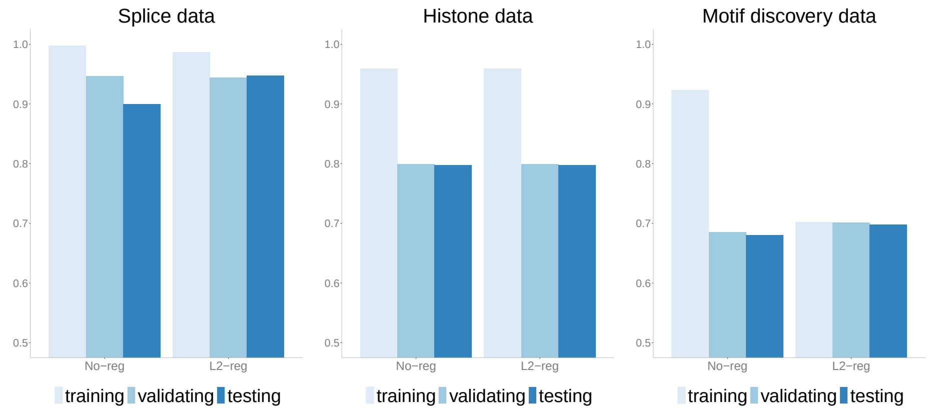

Figure 3 Accuracy of CNN-Zeng models with four 2D layer on three datasets of increasing size with and without regularization. The L2 regularization indeed reduces overfitting for the motif discovery data, but not for the histone data.

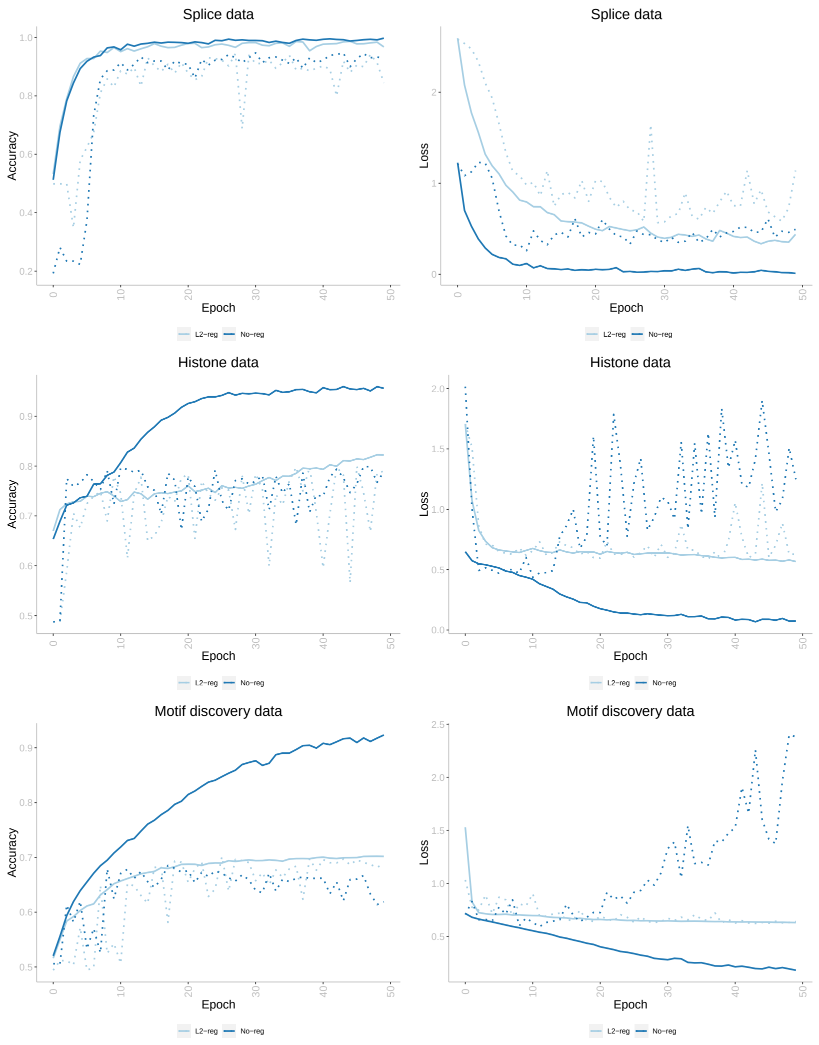

Figure 4 Learning dynamics for CNN-Zeng models with four 2D layer on three datasets of increasing size with and without regularization. Solid lines correspond to training accuracy/loss and dashed lines correspond to validating accuracy/loss. Colors represent no regularization or L2 regularization.

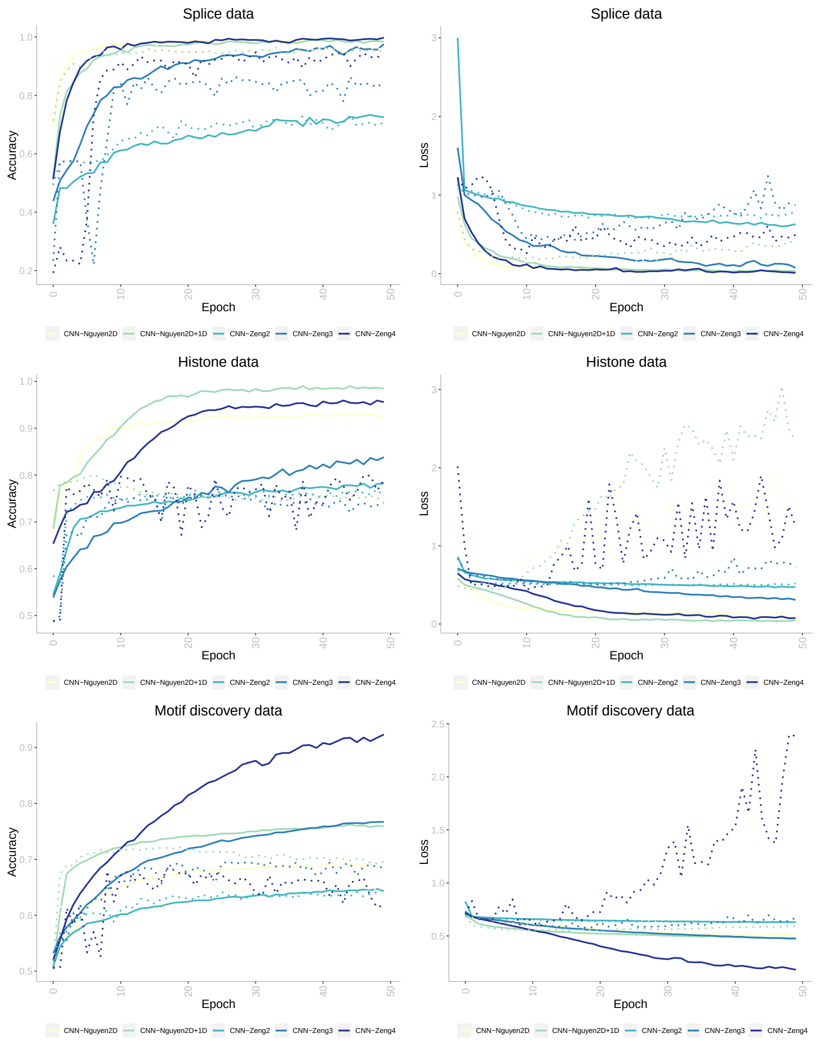

Figure 5 Learning dynamics for CNN models on different datasets. Solid lines correspond to training accuracy/loss and dashed lines correspond to validating accuracy/loss. Colors represent different models.

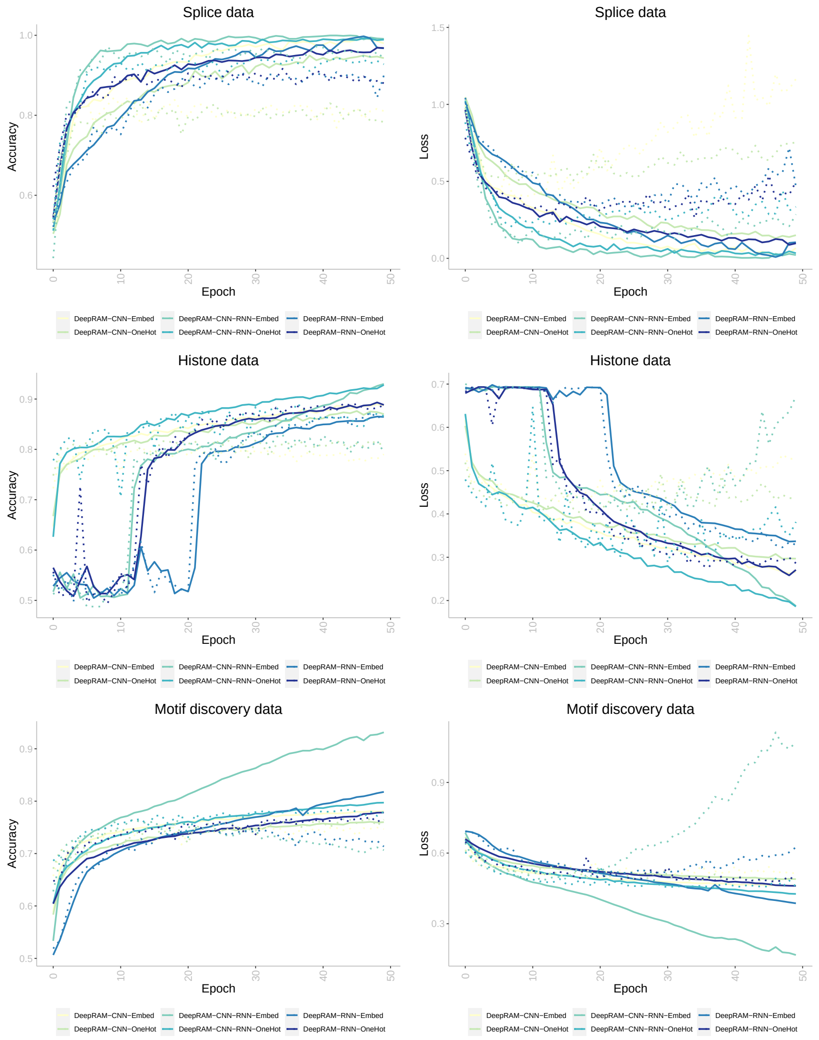

Figure 6 Learning dynamics for DeepRAM models on different datasets and different data embedding. Solid lines correspond to training accuracy/loss and dashed lines correspond to validating accuracy/loss. Colors represent different models and different data embedding.

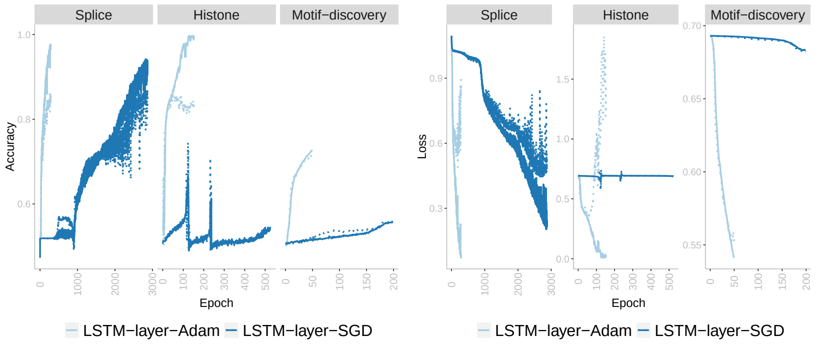

Figure 7 Learning dynamics for LSTM-layer model on different datasets and different optimizer. Solid lines correspond to training accuracy/loss and dashed lines correspond to validating accuracy/loss. Colors represent different optimizer.

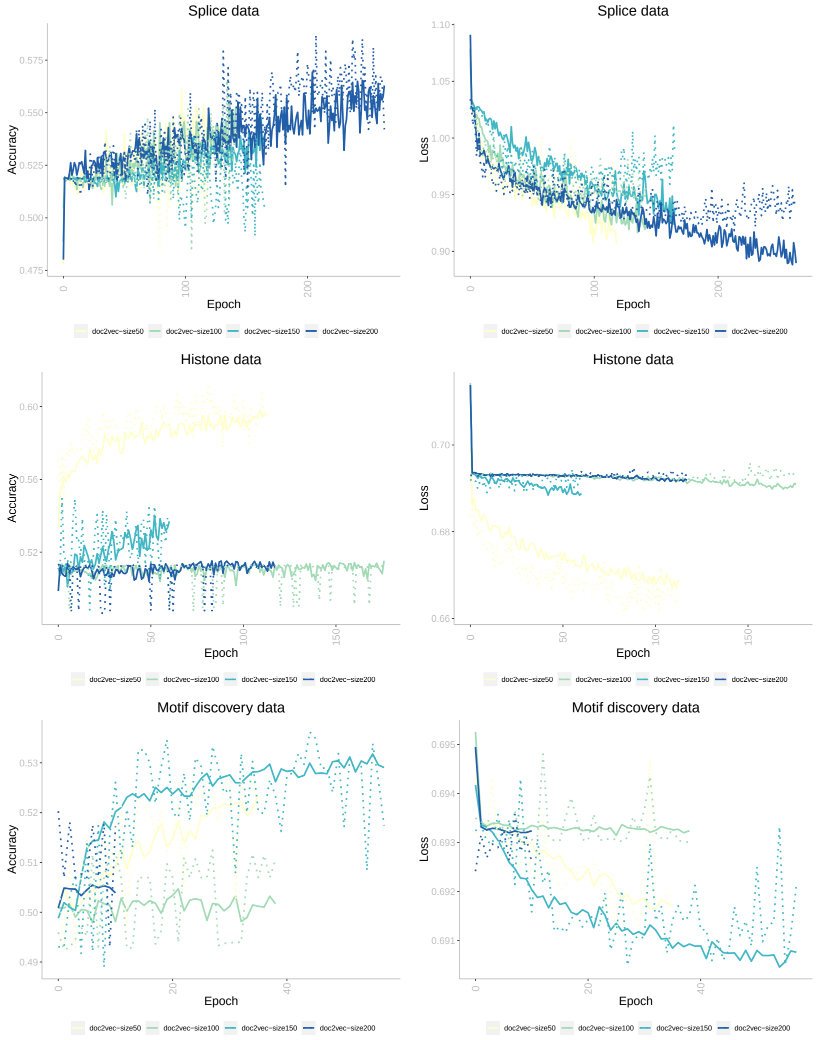

Figure 8 Learning dynamics for doc2vec+NN model on different datasets and different embedding sizes. Solid lines correspond to training accuracy/loss and dashed lines correspond to validating accuracy/loss. Colors represent different embedding sizes.

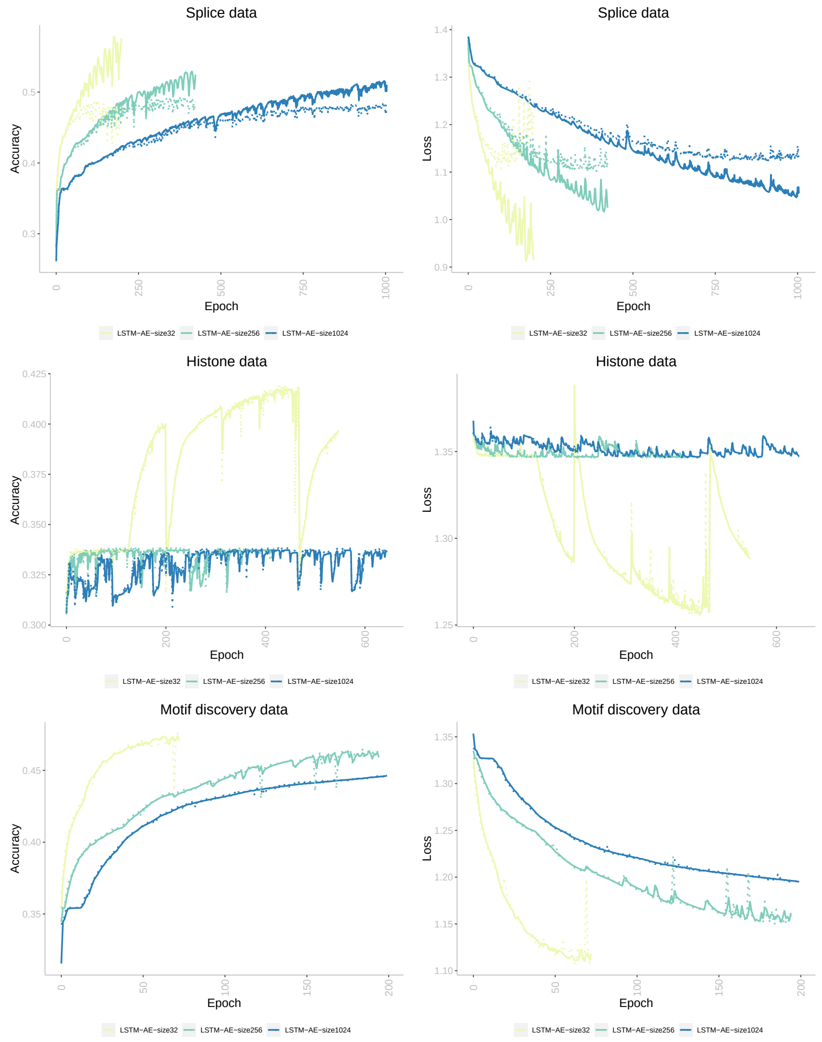

Figure 9 Learning dynamics for LSTM-AE+NN model on different datasets and different batch sizes. Solid lines correspond to training accuracy/loss and dashed lines correspond to validating accuracy/loss. Colors represent different batch sizes.
